# Evaluation of Efficacy and Safety Using Low Dose Radiation Therapy with Alzheimer’s Disease: A Protocol for Multicenter Phase II Clinical Trial

**DOI:** 10.3233/JAD-230241

**Published:** 2023-09-26

**Authors:** Dong-Yun Kim, Jae Sik Kim, Young-Seok Seo, Woo-Yoon Park, Byoung Hyuck Kim, Eun-Hee Hong, Ji Young Kim, Seong-Jun Cho, Hak Young Rhee, Aryun Kim, Keun You Kim, Dae Jong Oh, Weon Kuu Chung

**Affiliations:** a Department of Radiation Oncology, Kyunghee University Hospital at Gangdong, Seoul, Korea; b Department of Radiation Oncology, Soonchunhyang University Seoul Hospital, Seoul, Korea; c Department of Radiation Oncology, Chungbuk National University Hospital, Cheongju, Korea; d Department of Radiation Oncology, Seoul Metropolitan Government Seoul National University Boramae Medical Center, Seoul, Korea; e Radiation Health Research Institute, Korea Hydro & Nuclear Power Co Ltd., Seoul, Korea; f Department of Neurology, Kyunghee University Hospital at Gangdong, Seoul, Korea; g Department of Neurology, Chungbuk National University Hospital, Cheongju, Korea; h Department of Psychiatry, Seoul Metropolitan Government Seoul National University Boramae Medical Center, Seoul, Korea; i Workplace Mental Health Institute, Kangbuk Samsung Hospital, Sungkyunkwan University School of Medicine, Seoul, Korea

**Keywords:** Alzheimer’s disease, low-dose radiation therapy, protocol, randomized controlled trial

## Abstract

**Background:**

Alzheimer’s disease (AD), the most common cause of dementia, is a neurodegenerative disease resulting from extracellular and intracellular deposits of amyloid-β (Aβ) and neurofibrillary tangles in the brain. Although many clinical studies evaluating pharmacological approaches have been conducted, most have shown disappointing results; thus, innovative strategies other than drugs have been actively attempted.

**Objective:**

This study aims to explore low-dose radiation therapy (LDRT) for the treatment of patients with AD based on preclinical evidence, case reports, and a small pilot trial in humans.

**Methods:**

This study is a phase II, multicenter, prospective, single-blinded, randomized controlled trial that will evaluate the efficacy and safety of LDRT to the whole brain using a linear accelerator in patients with mild AD. Sixty participants will be randomly assigned to three groups: experimental I (24 cGy/6 fractions), experimental II (300 cGy/6 fractions), or sham RT group (0 cGy/6 fractions). During LDRT and follow-up visits after LDRT, possible adverse events will be assessed by the physician’s interview and neurological examinations. Furthermore, the effectiveness of LDRT will be measured using neurocognitive function tests and imaging tools at 6 and 12 months after LDRT. We will also monitor the alterations in cytokines, Aβ_42_/Aβ_40_ ratio, and tau levels in plasma. Our primary endpoint is the change in cognitive function test scores estimated by the Alzheimer’s Disease Assessment Scale-Korea compared to baseline after 6 months of LDRT.

**Conclusions:**

This study is registered at ClinicalTrials.gov [NCT05635968] and is currently recruiting patients. This study will provide evidence that LDRT is a new treatment strategy for AD.

## INTRODUCTION

The aging population is a global mega-trend, and the expected increase in the population with dementia places a heavy socioeconomic burden on most countries [1]. Approximately 50 million people worldwide have dementia, with nearly 10 million new cases occurring each year [1, 2]. Dementia is characterized by a marked loss of cognitive and emotional abilities and negatively affects daily functioning and activities [3, 4]. Alzheimer’s disease (AD) is the most common cause of dementia, a degenerative disease that results from extracellular and intracellular deposits of amyloid-β (Aβ) plaques and insoluble intracellular neurofibrillary tangles in the brain [5]. Despite numerous clinical studies investigating pharmacological approaches, only four drugs (donepezil, rivastigmine, galantamine, and memantine) have been available on the market to relieve symptoms in patients with AD [6]. Recently, aducanumab has been approved by U.S. Food and Drug Administration as the first mechanism-based fundamental disease-modifying drug for AD targeting Aβ plaques [7], but controversy over its efficacy continues due to ambiguous clinical results [8]. In addition, aducanumab remains inaccessible due to high burden of cost [8]. Furthermore, aducanumab has not yet been approved by the Korean Ministry of Food and Drug Safety, making it difficult for domestic patients to access it.

Meanwhile, as a non-pharmacological intervention, low-dose radiation therapy (LDRT) has gained attention after a case report described significant improvement in a patient with advanced AD who underwent computed tomography (CT) scans of the brain five times [9, 10]. Recently, several preclinical studies based on mouse models revealed a significant reduction in Aβ plaques with LDRT [11–13], and LDRT induced the upregulation of pre- and post-synaptic molecules in the brains of AD mouse models [14]. Moreover, a recent study showed that LDRT seems to reduce the levels of pro-inflammatory cytokines in animal models of AD [11, 14]; therefore, several pilot studies or clinical trials investigating the effect of LDRT on humans have been launched [15, 16]. Most recently, researchers at Virginia Commonwealth University (VCU) published positive pilot study results showing that four of five patients diagnosed with early AD experienced improved or stable cognition after treatment with LDRT [17]. However, the efficacy of LDRT has not yet been clearly demonstrated, and the optimal LDRT dose scheme for AD is still exploratory.

This study aims to explore LDRT for the treatment of patients with AD based on preclinical evidence and case reports. We will evaluate the safety and potential therapeutic effects of LDRT and explore the appropriate LDRT dose per fraction and schedule.

## MATERIALS AND METHODS

### Study design and registration

**Fig. 1:**
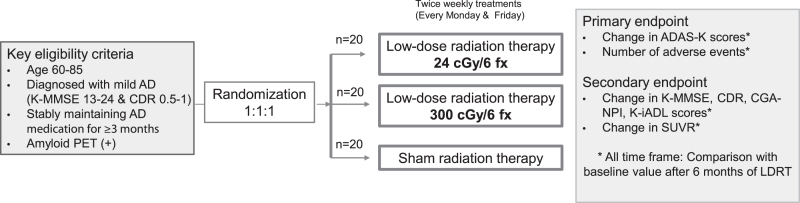
Schematic design of the clinical trial. AD, Alzheimer’s disease; K-MMSE, Korean-Mini-Mental Status Examination; CDR, Clinical Dementia Rating scale; PET, positron emission tomography; ADAS-K, Alzheimer’s disease assessment scale-Korea; CGA-NPI, Caregiver Administered-Neuropsychiatric Inventory; K-iADL, Korean Instrumental Activities of Daily Living; SUVR, standardized uptake value ratio; LDRT, low-dose radiation therapy.

This phase II multicenter prospective randomized controlled trial will evaluate the safety and efficacy of LDRT in the whole brain of patients with AD and determine the potentially applicable radiation dose. This trial is registered on ClinicalTrials.gov [NCT05635968]. Patients who are candidates for this study will be randomized into three treatment groups at a 1:1:1: ratio: 24 cGy/6 fractions of LDRT, 300 cGy/6 fractions of LDRT, or sham RT group (Fig. 1).

### Recruitment and eligibility criteria

This trial is conducted jointly by three institutions: Kyung Hee University Hospital in Gangdong, Chungbuk National University Hospital, and SMG-SNU Boramae Medical Center. Among the patients with AD who visit the Department of Neurology of each institution, those who meet the inclusion criteria for this study are referred to the Department of Radiation Oncology. In addition, with content approved by the Institutional Review Board (IRB), we are promoting patient recruitment through in-hospital publicity and advertisements in newspapers and subways.

The inclusion and exclusion criteria are presented in Table 1. All the following criteria should be satisfied for the patients to be included in the study: 1) age between 60 and 85 years; 2) diagnosed with probable AD dementia based on the new diagnostic criteria for AD outlined by the National Institute on Aging and Alzheimer’s Association; 3) stable maintenance of general AD drug treatment (donepezil, galantamine, rivastigmine, or memantine) for more than 3 months; 4) amyloid accumulation in the brain confirmed by amyloid positron emission tomography (PET); 5) mild AD (score range of 13 to 24 on the Korean Mini-Mental State Examination [K-MMSE] or 0.5 or 1 on the Clinical Dementia Rating scale [CDR]); 6) ability to perform cognitive function tests and imaging tests without help of others; and 7) accompanied by a guardian who provides information on the patient’s overall status, cognitive function, and functional changes. Patients who meet any of the exclusion criteria will be excluded from the study. The major exclusion criteria are 1) previous history of radiation to the brain; 2) previous history of malignancy diagnosed within 5 years from the time of screening or currently being treated; 3) cognitive decline associated with drugs or neurological or neurodegenerative conditions, not AD; and 4) clinically significant and unstable mental illness. The exclusion criteria are presented in Table 1.

**Table 1:** The inclusion and exclusion criteria for the clinical trial

Inclusion	Exclusion
Aged between 60 and 85 years	Previous history of radiation to the brain
Diagnosed with AD dementia based on the new diagnostic criteria for Alzheimer’s disease outlined by the National Institute on Aging and Alzheimer’s Association	History of seizure within the previous 10 years of the screening time
Stably maintaining the general AD drugs (donepezil, galantamine, rivastigmine, or memantine) for more than 3 months	Skin disease on the scalp
Amyloid accumulation in brain confirmed by Amyloid PET	Previous history of malignancy diagnosed within 5 years from the time of screening or currently being treated
Mild AD (score range of 13 to 24 on the K-MMSE or 0.5 or 1 on the CDR	Pregnancy or breastfeeding
Able to perform cognitive function tests and imaging tests	Subjects with cognitive decline associated with drugs or neurological / neurodegenerative conditions, not AD (i.e., drug abuse, vitamin B12 deficiency, thyroid dysfunction, stroke or other cerebrovascular conditions, Lewy body dementia, frontotemporal dementia, and head trauma).
Accompanied by a guardian who provides information on the subject’s overall status, cognitive function, and functional changes	Clinically significant, unstable mental illness (i.e., uncontrolled depression, schizophrenia, and bipolar disorder) within the last 6 months
Written informed consent was provided by the subject or the guardian to participate in this trial	Subjects whose brain MRI confirms evidence of: acute or subacute hemorrhage, prior macro hemorrhage (defined as > 1 cm in diameter, T2 sequence) or prior subarachnoid hemorrhage unless it can be documented that the finding is not due to an underlying structural or vascular abnormality, more than 4 microhemorrhages, cortical infarct (defined as > 1.5 cm in diameter, irrespective of anatomic location),>1 lacunar infarct (defined as > 1.5 cm in diameter), superficial siderosis, and history of diffuse white matter disease as defined by a score of 3 on the age-related white matter changes scale. Any finding that, in the opinion of the investigator, might be a contributing cause of the subject’s dementia, or pose a risk to the subject, or might prevent a satisfactory MRI assessment for safety monitoring are also included in the exclusion criteria
	Those who have other findings that are considered clinically important, and those who are judged by the researcher to be inappropriate for participation in this study.

AD, Alzheimer’s disease; PET, positron emission tomography; K-MMSE, Korean Mini-Mental State Examination; CDR, Clinical Dementia Rating scale; MRI, magnetic resonance imaging.

### Screening tests (Visit 1)

**Fig. 2:**
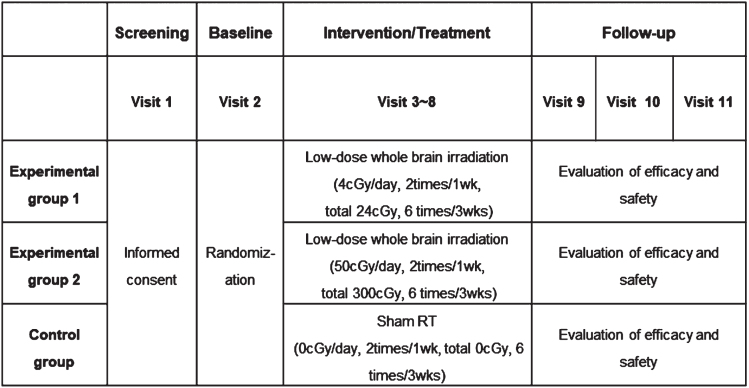
The overall progress scheme of the clinical trial.

The overall progress of this clinical trial is shown in Fig. 2. After voluntary consent of patients and guardians for this trial is obtained, screening tests will be performed to determine whether the participants meet the inclusion and exclusion criteria (Visit 1). Underlying disease, premedication, vital signs, neurological examinations, laboratory tests, amyloid PET, brain magnetic resonance imaging (MRI), and cognitive function tests will be performed. The baseline neurological examinations will be evaluated by a neurologist. Laboratory tests include complete blood cell count, blood chemistry examination, electrolytes, thyroid hormone (T3, T4, thyroid-stimulating hormone), genetics testing (apolipoprotein E genotyping), syphilis test (venereal disease research laboratory or rapid plasma reagin), serum Aβ and tau protein, and serum cytokines (interleukin-1 [IL-1], tumor necrosis factor-α, IL-10, and transforming growth factor-β). Amyloid PET involves daily quality control using Na-22/Ge-68 line sources to eliminate errors due to differences in amyloid PET specifications and protocols by institution. Amyloid PET images will be acquired at 90-min intervals following prolonged administration of flutemetamol 5 mCi using the standard protocol provided by the manufacturer of Vizamyl. In addition to visual readings, image readings will be quantified by measuring the standardized uptake value ratio (SUVR) of the target area. Brain MRI will be performed according to the imaging protocol of each hospital and saved in Digital Imaging and Communications in Medicine format. In the case of cognitive function tests, the following five tests will be performed: the Korea-Mini-Mental Status Examination (K-MMSE-2), CDR, Alzheimer’s Disease Assessment Scale –Korea (ADAS-K), caregiver administered-neuropsychiatric inventory, and Korean Instrumental Activities of Daily Living (K-iADL).

### Baseline and randomization (Visit 2)

Eligible patients will be assigned to three treatment groups (two experimental groups and one control group) by stratified block randomization using a random number table. The researcher will obtain consent and assign the screening number to each participant in order. After confirming eligibility, the participants will finally be enrolled, and a treatment group will be assigned based on a pre-generated random assignment table. Randomization will be performed on three treatment groups at a 1:1:1 ratio: 24 cGy/6 fractions of LDRT, 300 cGy/6 fractions of LDRT, or sham RT. After allocation, patients will undergo CT simulation of the whole brain using personalized immobilization devices. Treatment planning will be performed using three-dimensional conformal RT.

### Intervention/treatment procedures (Visits 3–8)

**Fig. 3:**
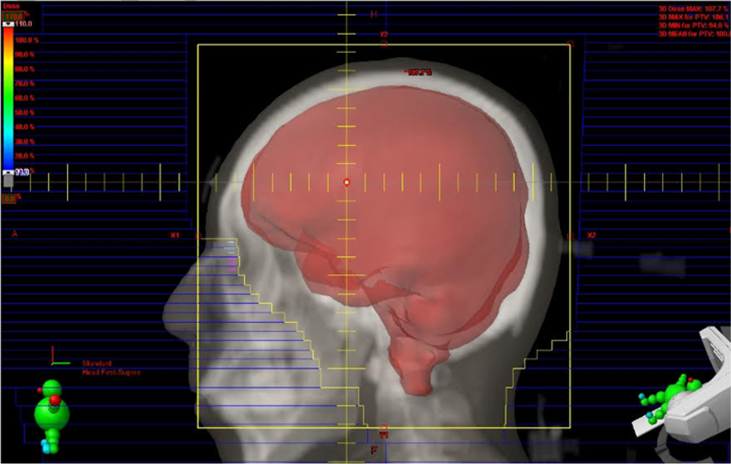
Target volumes for LDRT include the whole brain up to the C2 level to sufficiently cover the base of the skull.

Eligible patients will begin receiving interventions within 21 days of the screening tests. The experimental groups will be irradiated twice per week for 3 weeks (a total of six times) according to the radiation dose determined for each group, and the control group will be irradiated with sham irradiation. Twice weekly treatments are to be administered every Monday and Friday. A linear accelerator with 6 MV energy will be used to deliver whole brain irradiation to the patients assigned to the experimental groups. The target volume includes the whole brain at the C2 level to cover the skull base sufficiently (Fig. 3). The first experimental group will receive 4 cGy per fraction with a total of 24 cGy of LDRT. The second experimental group will receive 50 cGy per fraction, with a total of 300 cGy of LDRT. The sham irradiation group will be identical to the experimental group in all respects, including CT simulation, RT schedule, set-up procedure, gantry location, and in-room duration (approximately 20 min). Neurological examination, vital signs, and adverse events will be evaluated at every visit for the intervention.

### Follow-up (Visits 9–11)

All participants will visit the hospital approximately 1, 6, and 12 months after LDRT (visits 9, 10, and 11) to evaluate efficacy and safety. The examinations to be performed during the follow-up period are described in the Clinical Trial Schedule (Table 2).

**Table 2:** Overall clinical trial schedule and detailed examination tests for the participants

	Screening	Baseline	Intervention/Treatment						Follow-up		
Visit	V1	V2	V3	V4	V5	V6	V7	V8	V9	V10	V11
Week(s)	–3 w ∼ –1w	–1w	1w	1w	2w	2w	3w	3w	7w	27w	51w
Day(s)	–21 ∼ –1	–7 ∼ –1	1	5±3d	8±3d	12±3d	15±3d	19±3d	49±7d	189±7d	357±7d
Informed consent	•									
Basic information	•									
^Underlyingdisease^	•									
Premedication	•									
Vital sign	•	•	•	•	•	•	•	•	•	•	•
Neurological examinations	•	•	•	•	•	•	•	•	•	•	•
Laboratory tests^1^	•									•	•
serum amyloid-β & tau protein	•										•
Amyloid PET	•									•	•
*APOE* genotyping	•									
serum cytokines^2^	•									•	•
K-MMSE-2	•									•	•
CDR	•									•	•
ADAS-K	•									•	•
CGA-NPI	•									•	•
K-iADL	•									•	•
Brain MRI	•									•	•
Low-dose radiation therapy			•	•	•	•	•	•		
Randomization		•								
Assessment of adverse event			•	•	•	•	•	•	•	•	•

^1^Laboratory tests include complete blood cell count, blood chemistry examination, electrolytes, thyroid hormone test. ^2^Serum cytokines include Interleukin-1 [IL-1], tumor necrosis factor-α [TNF-α], IL-10, transforming growth factor-β. PET, positron emission tomography; *APOE*, apolipoprotein; K-MMSE, Korean-Mini-Mental Status Examination; CDR, Clinical Dementia Rating scale; ADAS-K, Alzheimer’s disease assessment scale-Korea; CGA-NPI, Caregiver Administered-Neuropsychiatric Inventory; K-iADL, Korean Instrumental Activities of Daily Living; MRI, magnetic resonance imaging.

### Outcome measures (primary and secondary endpoints)

The primary endpoint is the score change of ADAS-K (range 0–70, higher scores indicate a worse outcome) 6 months after baseline and the number of adverse events 6 months after baseline determined by the Radiation Therapy Oncology Group toxicity grading system. The amount of change will be evaluated as a valid response if it shows an improvement of≥5% from the baseline score.

The secondary endpoints include the score change of K-MMSE-2 (range 0–30, higher scores mean a better outcome), CDR (range 0–3, higher scores mean a worse outcome), CGI-NPI (range 0–144, higher scores mean a worse outcome), and K-iADL (range 10–33, higher scores mean a worse outcome) after 6, 12 months from baseline. Also, we will additionally assess the primary endpoint 12 months after baseline. Changes in the SUVR of amyloid PET scans in the frontal, parietal, lateral temporal lobe, anterior wedge, and posterior gyri will be evaluated. The evaluation of the valid response is the same as that of the primary endpoint.

Furthermore, the primary endpoint evaluation will be analyzed by three institutions, and an additional analysis will be conducted to determine whether there are any differences among institutions.

### Safety evaluation

In general, toxicities that occur when irradiating the whole brain in patients with brain tumors or brain metastases include headache, nausea, vomiting, fatigue, anorexia, scalp irritation/discoloration, hair loss, and abrupt cognitive decline. However, since the radiation dose that will be used in this study is much lower than used in patients with brain tumor (up to 5%), the aforementioned side effects are expected to be rare. Potential toxicities related to LDRT to the whole brain will be evaluated based on the Common Terminology Criteria for Adverse Events v6.0.

### Sample size calculation

This trial is intended to further evaluate the treatment effect of LDRT on AD, and it is difficult to calculate the appropriate effect size for the treatment and control groups. Therefore, the purpose of this study is exploratory, in the form of a pilot study to compare efficacy. Bonferroni correction will be performed, and a comparative, parallel, three-arm design will be adopted with 80% power and a 2-sided alpha of 0.05. Finally, 20 people per group, for a total of 60 people, is calculated as the number of samples considering the dropout rate.

### Statistical analysis

Baseline characteristics according to the three groups will be compared using Student’s unpaired *T*-test or Wilcoxon’s rank sum test for continuous values. For categorical variables, the chi-squared test or Fisher’s exact test will be performed. A paired *t*-test or Wilcoxon’s signed test will be performed to evaluate the changes after 6 months from baseline in each group.

### Ethics and dissemination

After approval by the Korean Ministry of Food and Drug Safety (No.1338), each institution obtained approval from its individual IRB. This study is registered with the Clinical Research Information Service [KCT0007550] and US National Library of Medicine (ClinicalTrials.gov) [NCT05635968]. After the final analyses are completed, the results will be published in international peer-reviewed journals and disseminated to the academic community and public.

### Composition and responsibilities

This study is conducted with the support of Korea Hydro & Nuclear Power, and all trial processes are managed and audited by the three institutions. The principal investigator is affiliated with Kyung Hee University Hospital at Gangdong.

## DISCUSSION

Despite significant advances in the pathophysiology of AD, it remains an incurable and debilitating neurodegenerative disease. As innovative strategies other than drugs have been actively tried [4], LDRT has emerged as a potentially new treatment modality for patients with AD. In previous preclinical studies conducted by our research team, LDRT demonstrated therapeutic effects on AD pathogenesis by reducing pro-inflammatory cytokines and Aβ plaques in a mouse model [12, 18]. In one study, the acute effects of LDRT significantly improved synaptic degeneration and neuronal loss in the hippocampus and cerebral cortex [12]. In another study, mice treated with LDRT showed significantly improved learning and memory skills compared with those in the sham RT group. In addition, pro-inflammatory cytokines decreased, and anti-inflammatory cytokines increased in the brain tissue of LDRT-treated 5xFAD mice [18]. These two previous studies based on AD animal models corresponded to a known pathogenesis of neuroinflammation [19], which causes the accumulation of IL-1-beta, IL-6, and transforming growth factor beta around the Aβ plaque [20]. Moreover, *in vitro* studies have reported that LDRT can contribute to amyloid clearance by breaking the H-bonds in amyloid plaques, opening the blood-brain barrier, and promoting the migration of monocytes. In addition, this is expected to help neuronal cell regeneration by affecting the activation of M2 microglia [21–23]. Based on the previous studies, clinical studies have been conducted to treat AD using LDRT (ClinicalTrials.gov Identifier: NCT02359864, NCT02769000, NCT03352258). Very recently, one study (NCT02769000) by VCU published the promising results of LDRT without safety issues. Five patients were enrolled in this pilot study, and they were treated with LDRT (2 Gy×5 fractions over 1 week). Four of 5 patients showed improved or stabilized cognitive function at 1 year after the treatment [17].

In the current study, we aim to evaluate the efficacy of LDRT in treating patients with AD who stably maintain the general AD drugs (donepezil, galantamine, rivastigmine, or memantine) for more than 3 months. This differs from previous NCT studies in that only patients taking AD medication were included. This is to confirm the possibility of LDRT as a combination therapy with conventional pharmacological interventions, rather than LDRT monotherapy. We will also include patients with mild AD (K-MMSE score of 13–24 or CDR score of 0.5–1). Patients with severe AD who are expected to have difficulty cooperating during screening tests or RT due to an excessive decline in cognitive function will be excluded from the study. Enrollment of patients with early-stage AD might be more difficult due to vague fears of irradiation for patients and caregivers; however, confirming the effectiveness of LDRT will be of great benefit to patients with early AD.

To determine the daily dose for the experimental groups, we referenced an experimental paper on radiation dose-dependent hippocampus apoptosis in adult mice [24]. In this study, dose-response curves were linear-quadratic, with a significant relationship between the appearance of cell death and irradiation dose in the range of 0–2 Gy. Furthermore, in a recent review article on low dose ionizing radiation affecting the immune system, Lumniczky et al. divided daily doses into three groups based on the following ranges: low-doses (<100 mGy), intermediate-doses (100 mcGy to 1 Gy), and high-doses (>1 Gy). High-doses mainly have immunosuppressive and pro-inflammatory effects, while low-doses have sustained immunomodulatory and anti-inflammatory effects [25]. Based on these facts, we adopted two experimental daily doses less than 1 Gy. The determination of the irradiation dose in experimental group 1 was based on a case report by Cuttler et al. [9], which showed remarkable cognitive improvement through a total of 5 trials of approximately 40 mGy per brain CT scan. The dose in experimental group 2 was determined according to Yang et al. [6]. In this study, mice will be irradiated with a total dose of 300 cGy (5 fractions×60 cGy) to prevent the accumulation of Aβ plaques and alleviate cognitive decline. A daily dose of 200 cGy, which is currently used in other ongoing clinical trials, will not be used in this study because there was no therapeutic effect in the LDRT study at a daily dose of 180 cGy conducted by our research team as a pilot study (NCT04203121). A recent study by Rogers et al. showed promising results using a daily dose of 200 cGy, but it was a pilot study with only five patients, so the reliability of the daily dose cannot be determined [17]. To rule out a placebo effect, the control group will receive sham irradiation. Therefore, in this study, two experimental groups will be used: 4cGy per fraction (a total of 24 cGy/6 fractions) and 50 cGy per fraction (a total of 300 cGy/6 fractions) to verify the treatment effect compared with that in the control group (sham RT).

As for the twice weekly regimen rather than a daily RT, it was related to the LDRT as a treatment for osteoarthritis (OA). Several studies have demonstrated the efficacy of LDRT in modulating immune function, and it is used in European countries to relieve the symptoms of uncontrollable OA [26–28]. The most common regimen is to perform LDRT two to three times a week [28]. The interfraction interval is based on research by Moon et al. [29]. After hippocampal dentata gyrus (DG) was irradiated with LDRT, the number of Ki-67-positive and doublecortin-positive cells in DG declined sharply between 0 and 24-h post-irradiation, which then increased gradually to the sham RT level between 3 and 14 days of post-irradiation. Therefore, we tried to set the interval between twice-weekly treatments to no more than 3 days. Given the pilot clinical study by Cuttler et al. in which four AD patients were treated with LDRT at two-week intervals each using a CT scanner [15], it is reasonable to widen the RT interval. Furthermore, given the biology of normal neurons in response to radiation, it is likely that spacing the treatments is more favorable for repopulation than daily treatments [16].

Meanwhile, in the efficacy analysis, an improvement of≥5% will be evaluated as an effective response. To date, as the clinical trials on the effects of LDRT on patients with AD are ongoing or partially understood, a decision must be made after consultation with a neurologist. In the case of existing AD medication, the improvement in neurological function is almost 0%, and it only slows the rate of cognitive deterioration [30]. Therefore, LDRT was conservatively estimated to have a therapeutic effect, even with only a 5% improvement.

In conclusion, this trial will provide notable evidence on the efficacy and safety of LDRT as a new treatment strategy for AD.

## Data Availability

Research data are stored in an institutional repository and will be shared upon request to the corresponding author.
